# The Impact of Consumers’ Dynamic Browsing Modes on the Effect of In-Feed Native Advertising

**DOI:** 10.3389/fpsyg.2022.842906

**Published:** 2022-03-07

**Authors:** Bangming Xiao, Hao Zhang

**Affiliations:** ^1^College of Economics and Management, Huazhong Agricultural University, Wuhan, China; ^2^School of Economics and Management, Hubei University of Technology, Wuhan, China

**Keywords:** in-feed native advertising, ad persuasion styles, dynamic browsing modes, self-agency, external agency, agency theory

## Abstract

As an emerging form of online display advertising, in-feed native advertising is increasingly employed in online news feed platforms. While many advertisers have largely embraced this new advertising format, the current research is full of controversy on whether the more native, the better the effect of in-feed native advertising. Based on recent studies on this emerging topic, the authors explore the effective in-feed native advertising persuasion strategies based on consumers’ dynamic online browsing modes. In study 1, the authors conducted an archived-data analysis (in co-operation with Baidu company). Results show that the match between in-feed native advertising persuasion style (implicit vs. explicit) and consumers’ real-time news feed browsing modes (convergent vs. divergent) can improve ad performance. In study 2, the authors further explained why consumers under different browsing modes respond differently (the mediation effect of self-agency vs. external agency) to specific in-feed native advertising persuasion. Our work explores the boundaries of agency theory from a dynamic perspective and helps advertisers conduct real-time and effective targeting strategies.

## Introduction

As a new form of online display advertising, in-feed native advertising is part of a more significant shift toward “native” advertising (Chatterjee and Zhou, 2021). Unlike banner advertising, which is usually designed as out-standing or even intrusive, the in-feed native ad features in the “un-distinguishable” approach and tend to embed itself in the consumers’ online browsing content (e.g., the news feed in Facebook, USA Today, Baidu, and TopBuzz) (Chung and Kim, 2021). According to recent statistics in eMarketer (2019), online advertisers spent $44 billion in 2019, with a projected $53 billion being spent in 2020.^1^ The idea behind the in-feed native ads, in general, is to integrate the ads into the non-ad news feeds so that the ads would be less annoying and therefore induce higher click rates from the consumers (Zimand-Sheiner et al., 2020; Chatterjee and Zhou, 2021). In practice, most advertisers follow the “native” approach and intend to make the persuasion in in-feed native advertising as implicit as possible (Wojdynski and Evans, 2016; Chatterjee and Zhou, 2021). A typical implicit persuasion simply mention a product or service and suggest how it meets consumer’s needs without explicit behavioral oriented messages such as “get it today!,” “you gotta have it!,” “have a try!” (Summers et al., 2016). However, it still lacks empirical proof on the effectiveness and mechanisms of such intuitive ad persuasion strategy in the in-feed native advertising context.

Unlike traditional display advertising, in-feed native advertising is embedded in the flow of news feeds. Consumers in such context adopt varied browsing modes as they navigate online content, and their cognitive states change accordingly (Ruiz and Sicilia, 2004; Huang et al., 2009; Zhang et al., 2014). Past research has demonstrated that people behave consistently with various cognitive states (Jung et al., 2014; Summers et al., 2016). These findings are consistent with agency theory, which proposes that the individual’s behavior is regulated from either internal or external stimulus according to their sense of agency (Engström and Elg, 2015). A large number of studies in this literature have identified two types of agency: self-agency and external agency (Levesque and Pelletier, 2003; Dholakia, 2006; David et al., 2008). The self-agency is found when consumers willingly enact a specific action and feel their actions emanate from their own values and interests (Engström and Elg, 2015). Therefore, non-intrusive messages are preferred for consumers in this cognitive state (Lee et al., 2016). On the other hand, the external agency is found when the consumer’s action is non-directed and oriented to navigational choices (Novak et al., 2003). It is also found that consumers in such cognitive states prefer more directed and explicit messages because they are “externally regulated” and have no or a low level of competence for a task (Engström and Elg, 2015). However, in the context of in-feed native advertising, little is known about how to identify consumers’ different needs for agency according to the consumers’ dynamic browsing modes.

To answer questions for the in-feed native advertising practice and to fill the void in the agency theory literature, our work aims to identify consumers’ different needs for agency according to their dynamic browsing modes and examine how the in-feed native advertising message should match consumers’ needs for self and external agency. We propose that the consumer’s need for self-agency vs. external agency is dynamic. Specifically, when consumers get interested in a specific topic and reinforce their perceptions on such topic by reading more similar and relevant content (a convergent browsing mode), they are motivated to achieve utilitarian or functional benefits (Björneborn, 2008; Ko, 2020), and their decisions making rely more on the self-agency and therefore response positively to implicit persuasion. When the consumers are not so “serious” in their online navigations or simply get tired of reinforcement reading and turn to experience among varied topics (a divergent browsing mode), they are motivated to achieve hedonic or experiential benefits (Nysveen et al., 2005) and their decisions making rely more on the external agency and therefore respond positively to the explicit persuasion.

Two studies were conducted to support this theorizing and demonstrate how the ad persuasion style (implicit vs. explicit) matched the consumers’ dynamic online browsing modes (convergent vs. divergent). The first study examined the matching effect between consumers’ dynamic browsing modes and ad persuasion styles. We co-operated with the news feed department in Baidu company (one of the largest news feed media platforms in China) and compiled a dataset of clickstreams and browsing histories from sampled consumers. A lab experiment was conducted in the second study to explore this matching effect’s process. Above all, we suggest that a real-time “fit-in” strategy between consumers’ dynamic browsing modes and ad persuasion styles can achieve better performance (i.e., higher CTR or positive ad attitude).

Our research contributes to the literature in several ways: First, while previous research has examined the role of agency based on consumers’ relatively stable characteristics (David et al., 2008; Engström and Elg, 2015), we focus on the different roles of self-agency and external agency based on consumers’ dynamic traits. (The identifying approaches are also discussed). Second, this research also bridges the two literature streams of agency and matching effects in persuasion. Specifically, we propose that the self-agency (external agency) mediate the match between the implicit persuasion and the consumer’s convergent (divergent) browsing mode. This form of matching holds promise for enhancing in-feed native advertising persuasion.

This research also has important practical implications for marketers. We propose a dynamic persuasion strategy based on consumers’ real-time browsing content computations. This semi-automatic approach (text-mining of online content combined with manual coding of ad message), which considers the matching between the persuasion style and consumer’s dynamic cognitive states, can help the in-feed native advertisers anticipate and manage their targeting strategies. While traditional targeting strategies are mainly based on consumers’ demographical (Joshi et al., 2011) or psychographic traits (Wang et al., 2019), our research shows that consumers’ real-time online behavioral information can also be leveraged to improve in-feed native advertising targeting strategies.

## Literature Review

### In-Feed Native Advertising

“In-Feed Native Ads,” also called “Sponsored Stories,” “Article-Style Native Advertisements,” typically exist on social media platforms that offer a steady stream of social news (Chatterjee and Zhou, 2021; Chung and Kim, 2021). Since the in-feed native ad is a recent phenomenon, academic research on this topic is currently at a nascent stage. Most prior research has focused on the unique characteristics of in-feed native advertising and their effects on ad performance from mainly the following two perspectives.

#### The Ad Design Perspective

In recent years, there has been an increasing amount of literature investigating the effects of in-feed native advertising design from mainly two elementary streams: the visual effect (Chatterjee and Zhou, 2021) and the content effect (Hwang and Jeong, 2019). Given specific ad content, the research focusing on the visual impact of in-feed native advertising tries to unveil the mechanisms of how varied display or layout strategies attract consumers’ attention and their aftermaths (Wojdynski and Evans, 2016; Kim et al., 2019; Aribarg and Schwartz, 2020; Chatterjee and Zhou, 2021). For example, Chatterjee and Zhou (2021) present an analytical model to identify conditions under which competing platforms choose sponsored content advertising over traditional display advertising. They show that two symmetric media platforms can choose visually different advertising strategies, leading to an asymmetric equilibrium outcome. Aribarg and Schwartz (2020) also focus on the visual effect and study how consumers respond to native vs. visually intrusive ads. They find that the native ad generates a higher click-through rate because it better resembles the surrounding editorial content. In contrast, less is known about how to organize the in-feed native ad content effectively. Among the few papers related to the content of the in-feed native ads, Hwang and Jeong (2019) claim that whether a sponsoring brand is mentioned in editorial content affects consumers’ responses to native advertising. They find that brand content has a negative effect on source credibility and message attitudes only when the quality of editorial content is low. Kim et al. (2017) reveal that the content of well-matched products and spokespersons leads to a positive evaluation of in-feed native advertising.

Although prior research on the ad content effect has shown “what” content should be included and its effectiveness, little is known about “how” to frame the content. In the context of in-feed native advertising, the minimal native space makes the ad narratives short, brief, and title-like messages (sponsored hyperlink listings) embedded in surrounding articles’ headlines (Aribarg and Schwartz, 2020) and leaves the minimal choice of creative ad content. Therefore, the question of “how” to frame the content has become increasingly important. This is also related to the recent work of intelligent advertising that features consumer-centered, data-driven, and algorithm-mediated communication (Li, 2019). However, optimizing such algorithm-mediated communication requires insights into the mechanisms underlying the effects of varied persuasion messages and robust field tests (Rodgers, 2021). Previous studies that have explored the impact of ad content are implemented primarily by online surveys (Wojdynski and Evans, 2016; Kim et al., 2017). Relatively little has been explored in the field settings where the content effect in principle could be tested empirically by millions of real-world users. Among a few, Lee et al. (2018) content-code 106,316 Facebook messages and study ad content (informative vs. emotional) association with user response. Although their study explains how varied elements in the in-feed native ads can trigger different consumer responses in a real-world setting, there is little established knowledge regarding how to frame them to fit the targeted consumers under varied browsing conditions.

#### The User Heterogeneity Perspective

Apart from the ad design factors, the equally essential factors of consumer heterogeneities are also heavily studied in the literature (Zarouali et al., 2017; Wang et al., 2019). There is a long history of literature on ad targeting based on consumer heterogeneity, such as the consumer’s demographical or geographical traits (Joshi et al., 2011). One criticism of this traditional targeting strategy is its static (even unethical) view of consumers’ attributes. For example, Wang et al. (2019) unveil how consumers’ gender and age moderate the association between the serial positions of in-feed native advertising and its effectiveness. Zimand-Sheiner et al. (2020) find that adolescents generally accept native advertising as a moral practice, moderated by their country of origin.

Recently, the stream of behavioral targeting literature has emerged and proved that the abundant data of consumers’ online browsing history can be leveraged to increase ad performance (Summers et al., 2016; Boerman et al., 2017). For example, to examine the underlying mechanism of behavioral targeting, Summers et al. (2016) carry out a series of experiments. They conclude that a behaviorally targeted ad act as a social label to impact users’ purchase intentions. Boerman et al. (2017) review the literature from the period and illustrate how the advertiser-controlled and consumer-controlled factors can explain users’ responses to online behavioral targeting ads. These studies have made an enormous leap forward from the traditional static view into the new era of dynamic ad targeting. They all suggest that the abundant behavioral data are useful to increase ad targeting efficiency (Edwards and Allenby, 2003; Park and Fader, 2004; Joshi et al., 2011). However, the underlying mechanism of previous behavioral targeting literature, e.g., the social identity theory, still focuses on consumers’ relatively stable traits (Summers et al., 2016). There is a lack of research into consumers’ unstable and frequently (or easily) changed attributes, such as their real-time cognitive states as they navigate through online content. The seminal work of Thompson and Hamilton (2006) has demonstrated that matching ad format to consumers’ information browsing modes can enhance ad effectiveness. And several lines of evidence suggest that consumers usually show different and significant information browsing modes, e.g., goal-directed vs. experiential browsing mode (Novak et al., 2003) or depth vs. breadth browsing mode (Huang et al., 2009). More importantly, previous research has shown that consumers do not dwell on just one information browsing mode. As they navigate through varied online content, their information browsing modes change accordingly (Jung et al., 2014).

Above all, we illustrate the literature on the effect of in-feed native advertising from the ad design perspective and user heterogeneity perspective and highlight our focus and contributions in Table 1.

**TABLE 1:** Selective literature review of In-feed native advertising.

Literatures	Ad design perspective		User heterogeneity perspective		Research method	Main variables
	Visual	Content	Static and historical attributes	Dynamic and real-time attributes		
Wojdynski and Evans (2016)	Yes	No	No	No	Online survey + lab experiment	Disclosure position Disclosure language
Harms et al. (2017)	Yes	Yes	No	No	In-depth expert interview	Brand prominence Message appeal ad context
Kim et al. (2017)	No	Yes	No	No	Online survey	Product type Spokesperson
Zarouali et al. (2017)	No	Yes	Yes	No	Online survey	Ad format Privacy concern Textual debriefing
Campbell and Evans (2018)	Yes	No	No	No	Online survey	Companion banner ad Disclosure Presence
Lee et al. (2018)	No	Yes	No	Yes	Modeling	Persuasive content Informative content
Kim et al. (2019)	Yes	No	No	No	Online survey	Ad type Ad placement
Wang et al. (2019)	Yes	No	Yes	No	Modeling + natural experiment	AD serial position
Hwang and Jeong (2019)	No	Yes	No	No	Online survey	Brand placement Content quality
Zimand-Sheiner et al. (2020)	No	No	Yes	No	Online survey	Persuasion knowledge Advertising ethics
Sahni and Nair (2020)	Yes	No	No	Yes	Modeling + filed experiment	Ad disclosure
Aribarg and Schwartz (2020)	Yes	No	Yes	No	Modeling + field experiment	Ad formats Native ad disclosure prominence
Chatterjee and Zhou (2021)	No	Yes	No	No	Modeling	Ad content
**This paper**	**Controls**	**Yes**	**Controls**	**Yes**	**Modeling +lab experiment**	**Ad message framing Dynamic browsing mode**

### Ad Persuasion Styles: Implicit vs. Explicit

As for the content design in advertising, a substantial body of research has focused on the feasibility and effectiveness of varied persuasion technologies (Lohtia et al., 1995; Homer, 2008; Hwang and Jeong, 2019). Considerable progress has been made to conceptualize perceived explicitness (or implicitness) and understand its effect on ad performance (McQuarrie and Phillips, 2005; Okazaki et al., 2010). In general, two mutually opposite persuasion styles have been explored in prior research: implicit vs. explicit persuasion style (Okazaki et al., 2010; Summers et al., 2016). The implicit persuasion only mentions the characteristics and attributes of the product or service and implies that it is worth buying (e.g., “DirecTV, all the sports you love, all in one place”) (Summers et al., 2016). Summers et al. (2016) propose that implicit persuasion requires the consumer to fill in a small gap between the ad message and their behaviors. In contrast, the explicit persuasion associates the characteristics and attributes of products with direct decision-making behaviors (e.g., All the sports you love are here, you gotta have DirecTV!) (Krishnamurthy, 2000; Packard and Berger, 2017). Packard and Berger (2017) point out that explicit endorsements are more persuasive because explicit endorsers are perceived to like the product more and have more expertise.

The literature stream of “hard vs. soft sell” in advertising has provided abundant evidence to illustrate the effects of different persuasions (Homer, 2008; Okazaki et al., 2010). In the thorough review, Okazaki et al. (2010) propose that the primary difference between hard and soft sell relies on the implicitness or explicitness of the persuasion message. While a typical hard-sell appeal is intended to explicitly induce actions such as buying, clicking, downloading, etc., a typical soft-sell appeal is intended to be less direct. It simply tells the informative news about a product and induces (implicitly) feeling and thinking toward the product (Homer, 2008). From an online survey of 550 U.S. adult consumers, Lee et al. (2016) find that ad non-intrusiveness is positively related to attitude toward and sharing intention of native advertising. However, there is also literature that suggests the opposite. For example, Lohtia et al. (1995) argue that both the content elements (e.g., the ad messages) and design elements (e.g., the interactivity, color, and animation) that are associated with direct and explicit persuasion are expected to elicit a more positive cognitive and affective response from the consumers since they explicitly associate the incentives and benefits with the induced actions (cognitive response) and gain more attention and emotional attachments (affective response). Krishnamurthy (2000) also argues that while advertisements are typically helpful in improving brand attitude or recognition, the final action (e.g., buying, sharing, and engagement) can be generated if the advertisements explicitly associate incentives or benefits (e.g., free offer, discounts, social approvals, etc.) with that specific action.

Although prior research has contradictory findings regarding the effectiveness of implicit vs. explicit persuasions in ad message content, it is not hard to notice that they all propose and validate their predictions based on the consumers’ specific characteristics (Lee et al., 2016; Wojdynski and Evans, 2016; Kim et al., 2019). As the consumers move around different psychological states, their attitudes or reactions toward implicit or explicit persuasion may vary accordingly (Novak et al., 2003; Park and Fader, 2004). Specifically, consumers who are goal-oriented or utilitarian-oriented rather than hedonic and experiential during online browsing may prefer implicit persuasion because it is less intrusive and suits the consumer’s psychological state as self-determined (Lee et al., 2016). In contrast, consumers who are hedonic and experiential oriented in online browsing may prefer explicit persuasion because it provides the external stimulus, decreases the difficulty of information processing, and suits the consumer’s psychological state of need for external inspirations, expectations, and experience (Jung et al., 2014).

### Consumer’s Dynamic Browsing Mode: Convergent vs. Divergent

Research on the consumer’s online information searching behavior has suggested that consumers show different and significant browsing modes as they navigate through online content (Ruiz and Sicilia, 2004; Huang et al., 2009; Zhang et al., 2014). A practical approach to identifying consumers’ real-time online browsing modes is to track their online browsing data and analyze it longitudinally. For a certain period, consumers may get interested in a specific topic and reinforce their perceptions on this topic by reading more similar and relevant content. This behavior meets the consumer’s conscious and explicit information needs typically based on specific problems and work tasks (Björneborn, 2008). For other times, consumers may not be so “serious” in their online navigations or get tired of reinforcement reading and are inclined to experience among varied topics. This behavior may reflect the consumer’s more subconscious, implicit, and muddled information that needs to be driven by pleasure, curiosity, and interest space (Björneborn, 2008). Inspired by Björneborn’s (2008) discussion on convergent and divergent information browsing, we apply the terms of convergent and divergent browsing mode to conceptualize the consumer’s online browsing behavior in the in-feed native advertising context.

In general, the convergent browsing mode is goal-directed, focused, and rational (Novak et al., 2003; Nysveen et al., 2005). Consumers under the convergent mode tend to focus more on content relevant to their interests (Björneborn, 2008; Ko, 2020). They will directly search or filter out relevant information to meet their utilitarian benefits (Novak et al., 2003). Thus, the contrast effect is more likely to occur since the boundary between the content of their interest and the external stimulus (e.g., advertising) is perceived as explicit, unambiguous, or impenetrable (Lee and Suk, 2010). In contrast, the divergent browsing mode is explorative, impulsive, and intuitive (Novak et al., 2003). Consumers under the divergent browsing mode tend to be “experiential-oriented” and focus more on the information that is more likely to raise their arousal level and simulate their unconscious desire (e.g., inspiration, experience, or relaxation) (Nysveen et al., 2005; Björneborn, 2008). They may rely more on their feelings and make decisions with a low level of effort (Nysveen et al., 2005). Thus, the assimilation effect is more likely to occur since the boundary between the content of their interest and external stimulus is undefined, ambiguous, or permeable (Lee and Suk, 2010; Ko, 2020). Drawing on previous literature on consumers’ online browsing modes as convergent vs. divergent, we summarize the distinctions between the two information browsing modes in Table 2:

**TABLE 2:** Distinctions between consumers’ browsing modes: Convergent vs. Divergent.

Dimensions	Consumers’ browsing modes		Related literatures
	Convergent	Divergent	
Motivations	Goal-directed	Experiential	Novak et al., 2003; Nysveen et al., 2005; Björneborn, 2008
	Extrinsic	Intrinsic	Novak et al., 2003; Ko, 2020
Information processed	Depth	Breadth	Huang et al., 2009
Benefits/values	Utilitarian	Hedonic	Novak et al., 2003
Psychological state	Cognitive	Affective	Ruiz and Sicilia, 2004
Response to stimuli	Assimilation effect	Contrast effect	Lee and Suk, 2010

Another critical yet less studied feature of consumers’ information browsing modes is its dynamic and reversal pattern (Jung et al., 2014). Rather than staying in just one particular information browsing mode, consumers usually fall into different information browsing modes dynamically and interchangeably (Park and Fader, 2004). It is supported by the “reversal theory,” which employs a bistability concept toward human behavior complexity (Jung et al., 2014). Unlike various personality theories that apply a more static view on human behaviors, the reversal theory addresses human behavior’s complexity based on bistable states, reversals, and hedonic tone (Jung et al., 2014). There is no necessarily particular order between the convergent and divergent information browsing modes. Whether the consumers fall into any specific information browsing mode or move around different information browsing modes is mainly decided by themselves (Park and Fader, 2004; Jung et al., 2014).

### Matching Between Ad Persuasion Styles and Consumer’s Browsing Mode

We turn to the behavioral science literature to unveil what happens when consumers navigate through online news feed under varied and reversible information browsing modes and why they respond differently to the specific in-feed native ad persuasion. In general, when performing an action, a person may view their behavior as either self-instigated (self-agency) or instigated in response to an inducement by an external agent (external agency) (Levesque and Pelletier, 2003; Dholakia, 2006; David et al., 2008). Previous research shows that the extent to which agency matters to consumers may vary across choice contexts (Botti and McGill, 2011; Bhattacharjee et al., 2014). Therefore, in the in-feed native advertising context, the consumer’s sense of agency is highly dependent on what information browsing mode they adopt when they navigate through the online news feed. Although not directly examining the relationship between consumers’ online browsing modes and agency, previous longitudinal studies on motivation have found that internal and external motivation can be temporarily related to or activated from the individuals’ cognitive structures (Levesque and Pelletier, 2003). For example, external events that allow people to feel autonomous and competent can foster internal motivation (Levesque and Pelletier, 2003). Therefore, when the consumers are under convergent browsing mode, they process the information consistent with their goals and effectively manage their perceptions (Novak et al., 2003; Björneborn, 2008), thus leading to the need for self-agency. In contrast, external events that do not support individuals’ basic needs for competence and autonomy have repeatedly been found to thwart efforts toward self-determination and lead to heteronomy in the self (Levesque and Pelletier, 2003). Therefore, when consumers are under divergent browsing mode, they usually do not bother or manage to control themselves and rely on the external agency (e.g., the exogenous news feed content) to provide them with inspiration, experience, or relaxation (Björneborn, 2008).

**FIGURE 1:**
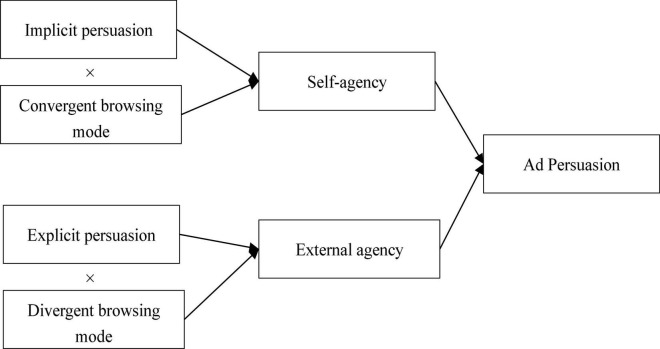
The conceptual framework.

We further suggest the possible links between the need for agency and ad persuasions. Specifically, the implicit persuasion will make the consumer perceive their act of ad-clicking due to their initiatives and thus suit the consumer’s need for self-agency (Ryan and Deci, 2006). In contrast, the explicit persuasion will make the consumer perceive their act of ad-clicking due to external suggestions and thus suit the consumer’s need for the external agency (Packard and Berger, 2017). With that being said, we propose that self-agency and external agency mediate the effect of the match between the consumer’s browsing modes and the ad persuasion styles on the consumer’s purchase intentions. Specifically, consumers in the convergent browsing mode need to freely choose and decide on the product they are interested in Novak et al. (2003) and Björneborn (2008). They prefer the implicit over the explicit ad persuasion because the implicit persuasion makes them feel that their decisions to click into the ads are self-induced and thus meet their needs for self-agency (Anker, 2020). When the consumer’s need for self-agency is enhanced, it leads to greater purchase intention (Bhattacharjee et al., 2014). Consumers in divergent browsing need an external agency to stimulate their unconscious desire (e.g., inspiration, experience, or relaxation) (Björneborn, 2008; Ko, 2020). They prefer the explicit over the implicit ad persuasion because the action-induced explicit persuasion saves effort from the consumer to process the information and provides inspirations and expectations (Novak et al., 2003; Lee and Suk, 2010). And when the consumer’s need for external agency is enhanced, it also leads to greater purchase intention (Anker, 2020). Above all, we propose the following hypothesis: (Figure 1).

**H1:** There should be a significant interaction between consumers’ real-time browsing behavior and the ad persuasion styles such that (a) consumers under convergent browsing mode will be more persuaded by implicit (vs. explicit) ad persuasion; and that (b) consumers under divergent browsing mode will be more persuaded by explicit (vs. implicit) ad persuasion.

**H2:** (a) The self-agency will mediate the effect of the match between consumers’ convergent browsing mode and implicit ad persuasion on consumers’ purchase intention; (b) and the external agency will mediate the effect of the match between consumers’ divergent browsing mode and explicit ad persuasion on consumers’ purchase intention.

## Study 1: Behavioral Data Analysis

### Data

The authors conduct this study in co-operation with the Baidu company in China. As the equivalent of Google in the U.S., Baidu is a Chinese multinational technology company specializing in internet-related services and products. They initiated their in-feed native advertising in September 2016 and have become one of the mainstream media platforms for in-feed native advertising. Our co-operation started in December 2017 with the support of the “intelligent advertising” program, in which the purpose is to explore the efficiency and effectiveness of dynamic ads generations and recommendations based on consumers’ behavioral data. After signing a confidentiality agreement, we were allowed to track the consumer behavior on the app for up to only 1 week. We agreed that the 7 days period was practical and reasonable in our research context because the average lifespan of an in-feed native ad was also a week. According to our pre-tests, on average, 357 consumers will generate over 2 million observations of behaviors (e.g., click, scroll, comment, etc.) throughout the 7 days, which has almost exceeded our computation capacity. For that matter, we agreed on a sample size of 357 randomly chosen consumers from the company’s server. The formal tracking began on December 24, 2017, and ended on December 30, 2017. Table 3 presents the description of our dataset from Baidu.

**TABLE 3:** Descriptive statistics of the dataset.

Data characteristics	Values	Definitions and descriptions
Sample size of users	357	Sample size of users
Window of observation	7 (days)	7 days of Baidu news app usages from sample users
# of impressions and clicks	1,422,465	Total number of views or clicks
Mean of impressions and clicks	569.2	The average number of views or clicks per user per day
Sample size of in-feed native ad	26,583	Sample size of ads
# Of impressions of sample ads	203,209	# of times of ads displayed
Ads clicked	13,980	Number of ads clicked
Implicit persuasion	17,810	Number of in-feed native ad persuasion coded as implicit
Explicit persuasion	8,068	Number of in-feed native ad persuasion coded as explicit
Impressions of implicit Persuasion	124,807	Number of implicit persuasion ads displayed
Impressions of explicit Persuasion	81,074	Number of explicit persuasion ads displayed
Clicks of implicit persuasion	3,744	Number of implicit persuasion ads clicked
Clicks of explicit persuasion	2,082	Number of explicit persuasion ads clicked

### Materials and Methods

#### Data Pre-processing

The pre-processing of our dataset mainly involves the screening and coding from the archived behavioral dataset across the research time window. Because a consumer’s browsing history consists of varied and unstructured data, and each observation of a consumer’s browsing history could be a mix of text, image, and even video, we employ dummy variables to indicate whether a specific record is text, image, or video. Each type of information has its own attributes being coded. For example, the text has the attributes of content (the complete characters in the text) and length (number of words). The image has the attributes of area (space each image occupies in the screen) and file size (indicating the fineness of an image). The video has the attributes of file size and duration (length of a video). Note that the image and video content are not furtherly coded on a pixel level for the following reasons. First, our research focuses on the text content in the in-feed native advertising, and we are inclined to control for the effects of image and video content. Second, most readers of news apps prefer to browse through the text content, for they probably have better options of apps to watch video content such as YouTube. According to the statistics in our dataset, only 7% of all the durations from all sampled consumers are spent on video content. However, with more sophisticated coding, future research can explore the image and video effects on consumers’ news browsing behaviors. We put that in our limitation and future research section. We employ the text-mining tool, the “JiebaR” package in R 3.5.0 (suitable to process Chinese characters), to word-segment and vectorize all the text contents in the consumer’s browsing history.

#### Measurements of Main Variables

After all the browsing histories of texts are word-segmented and vectorized, we can dynamically compute the main variables in our study based on these segmented words and their affiliated time stamps. Based on the definition of convergent vs. divergent browsing modes in the literature review part (Table 2), we apply the Herfindahl-Hirschman Index (HHI) (Lu et al., 2017) to represent the extent to which a focal consumer is under the convergent or divergent browsing mode. Specifically, we consider each unique word as a “firm” that comes from the whole “market” (i.e., the complete set of words in the consumer’s browsing history). Then a higher value of HHI generally represents a convergent browsing mode since some words account for relatively larger size (i.e., the frequency of each word) than others (a narrowed distribution of word frequency). A lower value of HHI generally represents a divergent browsing mode since the distribution of word frequency is somewhat flat in such case. Above all, we provide the measurement of the consumer’s dynamic browsing mode in the following equation.


(1) $${\text{BM}}_{i\,s}=\sum_{w=1}^{n\,s}{\left(W\,F_{w}\right)}^{2}$$

Where BM_*is*_ refers to the browsing mode of consumer *i* at session *s* (every 5 min of continuous online navigation as a browsing session). *WF*_*w*_ refers to each word’s frequency normalized by the total number of words (by text-segment from all the consumer’s browsing contents) during the browsing session *s*.

We adapted the definition from Okazaki et al. (2010) to code the ad persuasion style as implicit and explicit. Next, we hired 30 coders to rate the persuasion styles of all the ads in our dataset. Coders used a seven-point Likert scale (1 = strongly disagree, 7 = strongly agree) to rate the extent to which they agree with the following descriptions of the ads: (1) the ad simply mentions the product, brand or service attributes, characteristics, attitudes, and suggest how the product meet their needs; (2) the ad directly persuades me to make decisions and explicitly state that it is worth buying, owning, downloading, etc. All the coders were divided into two groups, and each group was assigned all the 26,583 pieces of ads while the students in the same group would split their tasks and complete their shares alone. The overall interrater agreement percentage was 0.73, and the kappa and tau correlations were 0.66 and 0.62, respectively. According to Multon (2010), a 70% level of agreement and a kappa of 0.50 are generally regarded as adequate. Finally, 8,773 pieces of ads are coded as explicit, and 17,810 pieces of ads are coded as implicit in our dataset. The following part will focus on the modeling process to explore the relationship between consumers’ browsing behaviors and ad responses.

#### Model Development

The model described in this section is based on a mix-effect Bayesian binary logit model (with the random-effect of the individual consumer to account for the endogeneity of ad-response from heterogenous consumers). In specific, whether a consumer *i* click on ad *j* is denoted as a dichotomous response *Y*_*ij*_, which comes from a Bernoulli distribution. We define the probability of user *i* clicking on ad *j* during a certain session as P(*Y*_*ij*_), note that a session is defined as a user’s continuous usage of the Baidu app. P(*Y*_*ij*_) is affected by a series of factors from both the individual user level and each ad’s design level. Therefore, users’ latent utility can be expressed as the following function:


(2) $$u_{i\,j}=\sigma \,P\,e\,r_{i\,j}+\beta \,B\,M_{i\,j}\,\gamma +\delta_{i\,j}+\mu \,\theta_{j}+\zeta_{i}+\varepsilon_{i\,j}$$

Whereε_*ij*_ is the idiosyncratic error that is independent across users, time, and ads. Assuming the type 1 extreme value distribution for this error, we can integrate it out and obtain the familiar logit form:


(3) $$\text{P}\,\left(Y_{i\,j}\right)=\frac{\exp\left(\sigma \,P\,e\,r_{i\,j}+\beta \,B\,M_{i\,j}+\gamma \,\delta_{i\,j}+\mu \,\theta_{j}+\zeta_{i}\right)}{1+\exp\left(\sigma \,P\,e\,r_{i\,j}+\beta \,B\,M_{i\,j}+\gamma \,\delta_{i\,t_{i\,j}}+\mu \,\theta_{j}+\zeta_{i}\right)}$$

$$=\frac{e\,x\,p\,\left(\sigma \,P\,e\,r_{i\,j}+\beta \,B\,M_{i\,j}+\sum_{m=1}^{5}\gamma_{m}\,\delta_{m\,i\,j}+\sum_{h=1}^{4}\mu_{h}\,\theta_{h\,j}+\zeta_{i}\right)}{1+e\,x\,p\,\left(\sigma \,P\,e\,r_{i\,j}+\beta \,B\,M_{i\,j}+\sum_{m=1}^{5}\gamma_{m}\,\delta_{m\,i\,j}+\sum_{h=1}^{4}\mu_{h}\,\theta_{h\,j}+\zeta_{i}\right)}$$

Here, the *t*_*ij*_ refers to the date the ad *j* is presented to user *i* during a certain session, and *t*_*ij*_ε{1,2…T} where T = 7 is the last date of our time-window; *Per*_*ij*_ is a time-invariant categorical variable and refers to the persuasion style of advertising *j* which is presented to user *i* and *Per*_*ij*_=1 stand for the ads encoded as implicit and *Per*_*ij*_=2 stands for the ads encoded as explicit; σ is the associated coefficient of *Per*; *BM*_*ij*_ is a variable describing the dynamic browsing mode of consumer *i* when facing ad *j*; β is the associated coefficient; δ_*ij*_ is a vector of time-variant variables controlling for the user level factors such as δ_1*ij*_ = total browsing time until ad *j* is presented to user *i*, δ_2*ij*_=total number of social news clicks until ad *j* is presented to user *i*, δ_3*ij*_=total number of ads clicks until ad *j* is presented to user *i*,δ_4*ij*_=total number of news impressions until ad *j* is presented to user *i*, and δ_5*ij*_=total number of ads impressions until ad *j* is presented to user *i*; γ_*m*_ is the associated coefficient of δ_*mij*_; θ_*j*_ is a vector of time-invariant variables controlling for the ad level factors such as θ_1*j*_=number of words in each ad text, θ_2*j*_ = size of each ad (size = width * length), θ_3*j*_=number of images presented in each ad, θ_4*j*_=days since each ad-push is initiated; μ_*h*_ is the associated coefficient of θ_*hj*_. We also include an individual fixed effect ζ_*i*_ that may influence the in-feed native advertising response. Including individual fixed effects here is important because it helps address the endogeneity issue. For instance, persistent individual preference may cause customers to be divergent/convergent browsing and prefer explicit/implicit advertising. Adding fixed effects can take out that concern. Above all, from a Bayesian perspective, given the observed data, the joint posterior distribution of all the parameters can be written as follows:


$$p\left(\sigma ,\beta ,\gamma ,\mu ,\zeta |Y_{i\,j}\right)=\propto p\left(Y_{i\,j}|\sigma ,\beta ,\gamma ,\mu ,\zeta \right)*p\left(\sigma ,\beta ,\gamma ,\mu ,\zeta \right)$$

$$p\left(Y_{i\,j}|\sigma ,\beta ,\gamma ,\mu ,\zeta \right)$$

$$\begin{array}{l} =\prod_{i=1;}^{i,j}{\left[\frac{\text{exp(}\sigma Per_{j}+\text{β}BM_{ij}+{\text{γδ}}_{ij}+{\text{μ}}_{h}{\text{θ}}_{j}+{\text{ζ}}_{i}\text{)}}{\text{1+exp(}\sigma Per_{j}+\text{β}BM_{ij}+{\text{γδ}}_{ij}+{\text{μ}}_{h}{\text{θ}}_{j}+{\text{ζ}}_{i}\text{)}}\right]}^{y_{ij}}.\\ j=1 \end{array}$$

(4) $${\left[1-\frac{\text{exp}\,\left(\sigma \,P\,e\,r_{j}+\beta \,B\,M_{i\,j}+\gamma \,\delta_{i\,j}+\mu_{h}\,\theta_{j}+\zeta_{i}\right)}{1+\text{exp}\,\left(\sigma \,P\,e\,r_{j}+\beta \,B\,M_{i\,j}+\gamma \,\delta_{i\,j}+\mu_{h}\,\theta_{j}+\zeta_{i}\right)}\right]}^{\left(1-y_{i\,j}\right)}$$

Where the *p*(*Y*_*ij*_|σ,β,γ,μ,ζ) is the likelihood function, and σ,β,γ,μ,ζ, all transferred to standardized regression coefficients, come from a normal distribution with vague and non-informative priors.

### Results

#### Parameter Estimates

To obtain posterior draws of the model parameters, we implement the MCMC (Markov chain Monte Carlo) procedure (Edwards and Allenby, 2003) from the “JAGS” package built-in R. We exclude the 5,000 burn-in draws and keep 1 in 20 (with randomized initial values for the MCMC chains) from extra 20,000 draws. All the descriptive statistics of variables are reported in Table 4. Table 5 reports the estimations of parameters in our proposed model.

**TABLE 4:** Descriptive statistics in study 1.

Main variables	Mean	*SD*	1	2	3	4	5	6	7	8
1. Ad CTR	0.03	0.02	-							
2. Browsing mode	1037.35	348.57	0.25**	-						
3. Browsing time	108.26	38.98	0.29**	0.03*	-					
4. Previous news clicks	16.61	3.96	0.18**	0.12**	0.14**	-				
5. Previous ads clicks	1.97	1.18	0.12**	0.23**	0.23**	0.17**	-			
6. Length of ad title	16.04	3.22	0.14**	0.26**	0.12**	0.16**	0.15**	-		
7. Ad news size	1.74	1.04	0.17**	0.17**	0.18**	0.02	0.03	0.11**	-	
8. Images number	2.89	1.78	0.05	0.11**	0.02	0.14**	0.16**	0.13**	0.04	-

*p < 0.05, **p < 0.01.

**TABLE 5:** Estimated posterior of parameters in ad-click model.

Dependent variable: probs of ad-click					
		
Independent variable			Main effect	Interaction effect	Full MODEL
			HDI percentiles (2.5%, 97.5%)	HDI percentiles (2.5%, 97.5%)	HDI percentiles (2.5%, 97.5%)
***Main effect***					
	In-feed native ad persuasion(0 for implicit, 1 for explicit)	σ	0.135 (–0.056, 0.147)	0.024 (–0.047, 0.078)	0.018 (–0.012, 0.037)
	Browsing mode (BM)	β	0.006 (–0.036, 0.015)	0.013 (–0.083, 0.053)	0.021 (–0.062, 0.136)
***Matching effect (Interaction)***					
	Per * BM			–0.107 (–0.154, –0.098)	–0.124 (–0.172, –0.105)
***Control variables (consumer Level)***					
	Browsing time	γ_1_			0.035 (0.031, 0.042)
	No. of previous news clicks	γ_2_			0.014 (–0.108, 0.117)
	No. of previous ads clicks	γ_3_			0.026 (0.021, 0.033)
	No. of previous news expressions	γ_4_			0.035 (–0.106, 0.058)
	No. of previous ads expressions	γ_5_			0.047 (–0.018, 0.065)
***Control variables (Ad Level)***					
	Length of ad title	μ_1_			0.016 (0.012, 0.019)
	Ad size	μ_2_			0.330 (0.310, 0.395)
	No. of images	μ_3_			0.214 (0.192, 0.243)
	Day 1 after ad push	μ_41_			0.023 (0.019, 0.036)
	Day 2 after ad push	μ_42_			0.017 (0.008, 0.029)
	Day 3 after ad push	μ_43_			0.270 (–0.008, 0.310)
	Day 4 after ad push	μ_44_			0.002 (–0.037, 0.014)
	*R* ^2^		0.13	0.17	0.25
	Δ*R*^2^		0.11	0.16	0.23
	–2LL		–23981.876	–16021.741	–13747.127
	Wald		1004.289***	1396.128***	1761.331***
	AIC		38617.278	30087.957	27709.018

***p < 0.001.

Table 5 shows that the main effect of ad persuasion style as implicit or explicit alone is insignificant. The consumer browsing mode is also insignificant (σ=0.018, β=0.021, and the zero fall within the HDI percentiles of these estimated posteriors). Besides, the main effect model has a relatively lower model fit level (adjusted *R*^2^ = 0.11). When the interactions between ad persuasion style and consumer browsing mode are considered, the predictive level of the model is increased. The interaction itself is significant (adjusted *R*^2^ = 0.23, Per*BM = –0.124), which indicates that the effect of explicit persuasion on ad-click probability is enhanced when the user is under the divergent browsing mode, and the effect of implicit persuasion is enhanced when the user is under the convergent browsing mode. Study 1 also considers the effects of user lever and ad level factors. Specifically, on the user level, the total browsing time and number of previous ad clicks have a significant impact on ad-click probability (γ_1_=0.035;γ_3_=0.026); while on the ad level, the number of words in the ad text, the size of the ad, and the number of images in the ad have significant effects on ad-click probability (μ_1_=0.016;μ_2_=0.330;μ_3_=0.214). Besides, the ad-click probability will decrease after the ad push is initiated. The time is no longer significant after it exceeds 3 days of the ad push (the coefficient of day 4 after the ad push is insignificant).

The data analysis based on the sampled server data reveals the relationship between the persuasion style, consumer browsing mode, and ad-click probability. But the results from study 1 could involve a serious endogeneity problem, and the mechanisms driving these results are still unknown. Therefore, we conduct a lab experiment in study 2 to explore the possible mechanisms underlying the results from study 1 and to solve the potential endogeneity problem in study 1.

## Study 2: Laboratory Experiment

Study 1 has empirically shown that the matching (interaction) between ad persuasion type and consumer’s real-time browsing mode can significantly improve advertising effectiveness. Study 2 is a designed laboratory experiment to explore the proposed mechanism (***H2a*** and ***H2b***) by directly measuring the self-agency and external agency. After manipulating participants into different conditions, we tested whether the self-agency and external agency mediated the relationship between the match of consumers’ real-time browsing modes and the in-feed native ads persuasion styles and the ad effectiveness.

### Materials and Methods

#### Pilot Study 1: Manipulation of Ad Persuasion Styles

Pilot study 1 was conducted to find an appropriate product to be used in the main study and confirm the manipulation of ad persuasion styles. According to Johanson and Brooks (2010), parameter estimates and confidence intervals are considered relatively high quality when the number of participants reaches the range of 30–50. In case of the absence of participants, we initially recruited 60 participants who were chosen randomly from a large university in China. Finally, 52 of them agreed to take part in the study. First, the participants rated their familiarity with six product categories for outdoor traveling. We selected sports watches as the stimuli because participants in our sample pool were familiar with them. Second, we created a fictitious brand of sports watches (“Narona”) and manipulated persuasion through ad messages. Ad format design was similar to those seen on the Baidu news feed platform, and the common image across the ads was of a sports watch without any brand information. To test the clarity of this distinction, we gave the participants the following definitions: “*Implicit persuasion ads*..simply mention the product, brand or service attributes, characteristics, attitudes, and suggest how the product meet their needs”; “*Explicit persuasion ads*.. directly persuade consumers to make decisions and explicitly state that it is worth buying, owning, downloading, etc.” Participants rated eight randomly ordered pairs of implicit persuasion ad (e.g., “All the sports watches you love, all in one place!”) and explicit persuasion ad (e.g., “All the sports watches you love here, you gotta have it!”) (1 = implicit persuasion ad, 4 = neutral, 7 = explicit persuasion ad). Compared to the scale midpoint, they reliably categorized both implicit persuasion ad [*M* = 2.73; *SD* = 0.69, *t*(52) = –13.28, *p* < 0.001] and explicit persuasion ad [*M* = 5.23; *SD* = 1.21, *t*(52) = 7.31, *p* < 0.001]. This study also verified that the participants did not recognize the brand of the watch.

#### Pilot Study 2: Manipulation of Browsing Modes

We ran pilot study 2 to ensure the successful manipulation of browsing modes. We initially recruited 60 participants who were chosen randomly from a large university in China. Finally, 3 of them have not completed their browsing task in the study. In the convergent browsing mode condition, participants were asked to imagine their upcoming trip to the Sichuan-Tibet line and then browse the news feed in Baidu. The participants were told to focus on the in-feed news related to the Sichuan-Tibet line tourism and draw up an itinerary template for this trip. In the divergent browsing mode condition, participants were asked to browse under the tourism column without explicitly stating which trip line they should focus on. To ensure that most participants had entered into the specific mode of information browsing, participants were asked to spend about 5 min to complete the task. Following past theorizing on consumers’ information browsing modes, we measured participants’ convergent browsing mode with two items (“I’m browsing information of similar topics,” “I feel very clear about my goals”; 1 = strongly disagree, 7 = strongly agree; Cronbach’s α = 0.94), and the divergent browsing mode with two items (“I’m browsing information of different fields or topics,” “I explore without a specific goal in mind”; 1 = strongly disagree, 7 = strongly agree; Cronbach’s α = 0.95). ANOVAs on these measures indicated that the convergent and divergent browsing instructions were successful. The convergent instructions generated more convergent processing mode significantly (*M* = 4.9) than the divergent browsing mode instructions [*M* = 3.3, *t*(1, 57) = 4.43, *p* < 0.001], and the divergent browsing mode instructions generated significantly more divergent processing (*M* = 5.0) than the convergent instructions [*M* = 3.25, *t*(57) = 4.81, *p* < 0.001].

#### Procedure

We initially recruited 240 participants who were chosen randomly from a large university in China for extra credit. Participants were randomly assigned to a 2 (ad persuasion style: implicit vs. explicit) × 2 (consumer browsing mode: convergent vs. divergent) between-subjects design. Finally, 235 of them (56.4% female, Mage = 22.8 years) agreed to take part in the study. The statistical power was computed using G*Power 3.1 software (Faul et al., 2009). For four groups, a total sample size of 235, an effect size (f) of 0.25, and a significance level of 0.05, the achieved power was 0.95, which is above the standard of 0.80. Participants were randomly assigned to a 2 (ad persuasion style: implicit vs. explicit) × 2 (consumer browsing mode: convergent vs. divergent) between-subjects design. Each participant was given a folder containing the news feed browsing mode instructions, a print ad for the watch, and a question booklet. First, we introduced the participants to different conditions of news feed browsing and asked them to spend about 5 min completing the task. After completing the browsing task, participants answered some filler questions to measure their browsing modes. Next, they were asked to evaluate the advertisement for Narona’s Watch. The only difference between the two conditions was the message used in the pretest (“All the sport watches you love, all in one place!” vs. “All the sport watches you love here, you gotta have it!”). Next, the participants indicated their purchase intention (“How likely are you to purchase this product?” 1 = not at all likely, 7 = very likely). Finally, they completed the underlying process measures. These statements directly follow the definition of perceived agency as the feeling that an outcome has emanated from the self or a force external to the self (David et al., 2008). They evaluated their perceptions of self-agency using three items (“I feel as if I own this choice of sports watch,” “This choice of a sports watch is an expression of my self-determination,” “I feel that I endorse this choice of sports watch”; 1 = strongly disagree, 7 = strongly agree; *a* = 0.92). The other three items were used to measure their perceptions of external agency (“The advertisement indicates that I’m the kind of person who would like sports watch,” “This advertisement indicates that I should have a sports watch,” “I am supposed to do it as advertised”; 1 = strongly disagree, 7 = strongly agree; *a* = 0.93). Twenty-two participants were excluded from the analysis since these tasks were incomplete during the experiment.

### Results

#### Manipulation Check

A one-way ANOVA revealed that participants in the convergent condition indicated higher convergent level than the divergent browsing condition [M_*convergent*_ = 5.45, *SD* = 0.61; M_*divergent*_ = 3.00, *SD* = 1.14; *F* (1, 209) = 395.94, *p* < 0.000, Cohen’s d = 2.68], and participants in the divergent browsing condition indicated higher divergent browsing level than the convergent condition [M_*divergent*_ = 5.42, *SD* = 0.89; M_*convergent*_ = 2.89, *SD* = 0.73; *F*(1, 209) = 514.81, *p* < 0.000,Cohen’s d = 3.11]. A one-way ANOVA on perceptions of advertising persuasion style revealed that the participants in the implicit persuasive condition were perceived as less persuasive than the explicit condition [M_*implicit*_ = 5.61, *SD* = 0.73; M_*explicit*_ = 2.63, *SD* = 0.53; *F*(1, 209) = 1175.674, *p* < 0.000, Cohen’s d = 4.67], and the participants in the explicit condition was perceived as more persuasive than the implicit condition [M_*explicit*_ = 5.27, *SD* = 0.89; M_*implicit*_ = 3.14, *SD* = 1.41; *F*(1, 209) = 172.99, *p* < 0.000, Cohen’s d = 1.81].

#### Purchase Intention

**FIGURE 2:**
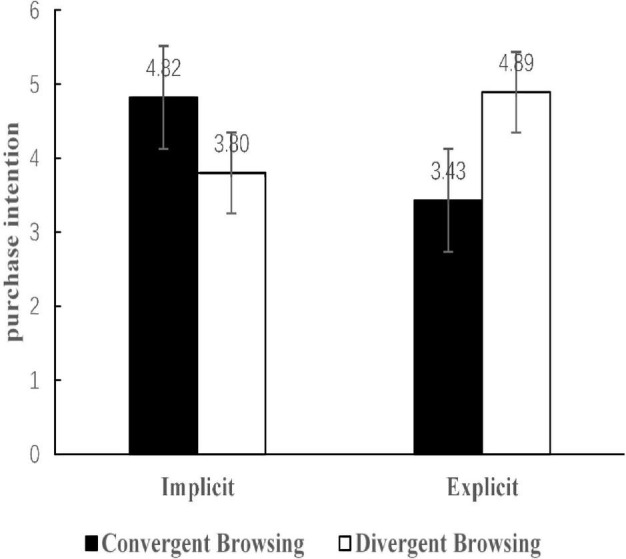
The interaction between ad persuasion styles and consumers’ browsing modes.

As expected, an ANOVA did not find a significant main effect of either ad persuasion style (*ps* > 0.31) or information browsing mode (*ps* > 0.14). However, it revealed a significant interaction effect of ad persuasion style and information browsing mode [*F*(1, 209) = 73.59, *p* < 0.001,η^2^ = 0.26]. *Post hoc* contrasts revealed that in the convergent browsing mode condition, consumers form a higher purchase intention toward the implicit than the explicit persuasion ad [M_*implicit*_ = 4.82, *SD* = 0.86; M_*explicit*_ = 3.43, *SD* = 0.91; *F*(1, 112) = 26.71, *p* < 0.001, η^2^ = 0.11], supporting ***H1a***. Conversely, in the divergent browsing mode condition, consumers form a higher purchase intention toward the explicit than the implicit persuasion ad [M_*explicit*_ = 4.89, *SD* = 1.02; M_*implicit*_ = 3.80, *SD* = 1.41; *F*(1, 97) = 54.88, *p* < 0.001, η^2^ = 0.19], consistent with ***H1b***. These findings support ***H1*** (see Figure 2). The fact that the effectiveness of implicit or explicit persuasion is significant only in certain information browsing modes provide further evidence for the importance of matching ad persuasion style to the consumer’s information browsing mode.

#### Mediation Analysis

We tested the hypotheses regarding the mediating roles of self-agency and external agency. A 2 (ad persuasion type) × 2 (consumer browsing mode) multivariate analysis of variance (MANOVA) on perceived self-agency and external agency revealed only an interaction effect of ad persuasion style and consumer’s browsing mode [for self-agency, *F*(1, 209) = 70.29, *p* < 0.001, η^2^ = 0.25; for external-agency, *F*(1, 209) = 109.39, *p* < 0.001, η^2^ = 0.34], but neither a main effect of ad persuasion style or consumer’s browsing mode (*ps* > 0.09). A mediated moderation analysis with ad persuasion style as the independent variable (0 = implicit, 1 = explicit), self-agency and external-agency as parallel mediators, consumer’s browsing mode (0 = convergent, 1 = divergent), and purchase intention as the dependent variable (Preacher and Hayes, 2004, Model 8; 5,000 Bootstrapped samples) shows that consumer’s browsing mode moderates mediation by self-agency and external-agency.

Conditional indirect effects show that when consumers are under convergent browsing mode, self-agency mediates the relationship between ad persuasion style and purchase intention (indirect effect = 0.68, *SE* = 0.16,99%CI = [0.287, 1.128]), but external-agency does not (indirect effect = –0.22, *SE* = 0.09, 99%CI = [–0.0122, 0.447]. For the self-agency path, the interaction between implicit (vs. explicit) persuasion ad and convergent (vs. divergent) browsing mode enhances the perception of consumer self-agency (β = 2.24, *SE* = 0.28, *t*(213) = 8.38, *p* < 0.001), which in turn promotes increased consumer intent to purchase [β = 0.35, *SE* = 0.62, *t*(213) = 5.62, *p* < 0.001]. These results indicate that in the convergent condition, perceived self-agency mediates the effects of ad persuasion style and purchase intention, whereas perceived external agency does not. Thus, ***H2a*** was supported.

When consumers are under divergent browsing mode, external-agency mediates the relationship between persuasion type and purchase intention (indirect effect = –1.22, *SE* = 0.18, 99%CI = [–1.758, –0.784]), but self-agency does not (indirect effect = 0.11, *SE* = 0.08, 99%CI = [–0.338, 0.064]. For the external-agency path, the interaction between explicit (vs. implicit) persuasion ad and divergent (vs. convergent) browsing mode enhances the perception of external agency [β = 2.32, *SE* = 0.22, *t*(213) = 10.46, *p* < 0.001], thereby increases the purchase intention [β = 0.62, *SE* = 0.08, *t*(213) = 8.23, *p* < 0.001]. These results indicate that in the divergent browsing condition, perceived external agency mediates the effects of ad persuasion style and purchase intention, whereas perceived self-agency does not. Thus, ***H2b*** was supported.

## General Discussion

Collectively, two studies support our theorizing regarding the match between the consumer’s browsing mode (convergent vs. divergent) and in-feed native advertising persuasion strategies (implicit vs. explicit). Specifically, in study 1, we use observational data obtained from Baidu to document consumers’ browsing-mode-specific heterogeneous responses to in-feed native advertising. After we control the ad level and consumer level heterogeneities, we find that a match between the persuasion style and consumers’ information browsing modes leads to a higher probability of clicking through the in-feed native advertising. In study 2, a follow-up lab experiment is conducted to shed light on the mechanisms underlying these matching effects and revealed that consumers’ need for agency drives the effect of the match. Mediational evidence demonstrates that self-agency works as a bridge between consumers in convergent browsing mode and their responses to implicit persuasion. In contrast, the external agency works as a bridge between consumers in divergent browsing mode and their responses to explicit persuasion.

### Theoretical and Managerial Contributions

Our findings offer several important theoretical contributions. First, previous research has shown that the sense of agency is central to human self-consciousness (David et al., 2008), and several studies have identified self-agency and external agency (Levesque and Pelletier, 2003; Dholakia, 2006). However, most of the studies discussed in this area explore the antecedents of the sense of agency from the individual’s relatively stable psychological traits. For example, the psychological needs of competence, autonomy, and relatedness are antecedents of self-agency (Engström and Elg, 2015), while the affective states of anxiety and tension are found to be associated with the external agency (Levesque and Pelletier, 2003). The current research enriches the understanding of agency and identifies self-agency and external agency from a dynamic context. Specifically, by leveraging the consumer’s real-time online browsing contents, we identify two information browsing modes (convergent vs. divergent) and show that the convergent information browsing mode can drive the consumer’s need for self-agency while the divergent information browsing mode can drive the consumer’s need for external agency. Our results also add to a growing number of studies exploring how consumers’ online behavior can affect their self-concept (Lee and Suk, 2010; Summers et al., 2016). Second, this research also bridges the two literature streams of agency theory and matching effects in persuasion. A considerable amount of research in advertising has demonstrated the effects of a match on persuasion and has examined various mediating processes in these relationships (Kim et al., 2017). We identify a novel process of self-agency and external agency based on the findings concerning the priming effect of agency on consumer behavior (Engström and Elg, 2015). Furthermore, most prior research investigating the matching effects in persuasion mainly focuses on the consumer’s stable traits. Such as the match between consumers’ demographical and geographical traits and the advertising contents (Joshi et al., 2011). This research focuses on consumers’ dynamic traits. The results show that the effectiveness of the in-feed native advertising persuasion is dynamic in nature and highly dependent on consumers’ need for self vs. external agency while they navigate through online content. These findings extend our understanding of the literature on agency and match effects in persuasion from a dynamic perspective.

This research also has timely and important implications for in-feed native advertisers. First, despite the prevalent use of native advertising in the news media (Wojdynski and Evans, 2016), questions remain about how native advertising is perceived and evaluated by consumers. In-feed native advertising has gained popularity because it is less intrusive and irritating (Hwang and Jeong, 2019). In practice, most advertisers intend to make the persuasion in in-feed native advertising as implicit as possible (Wojdynski and Evans, 2016; Chatterjee and Zhou, 2021). However, our results show that consumers under convergent (vs. divergent) browsing mode will be more persuaded by implicit (vs. explicit) ad persuasion. These findings can help the in-feed advertisers better anticipate and manage their targeting strategies. Second, as traditional ad targeting strategy relies heavily on the relatively static customer segmentation (Joshi et al., 2011), our results show a more dynamic and effective persuasive system based on the computations of the consumers’ real-time browsing content. For instance, with the help of text-mining techniques (Joshi et al., 2011), current research data demonstrates that consumers’ real-time online browsing contents can be analyzed to indicate their cognitive states, which in turn determines the effectiveness of the in-feed native advertising. In practice, with the help of more advanced text-mining and machine learning techniques (Lee et al., 2018), the in-feed native advertising contents can be automatically rephrased and adjusted to match consumers’ real-time cognitive states and increase the overall effectiveness of ad persuasion.

### Limitations and Future Research

This research also has several limitations: First, while we focus only on the texts in the in-feed native advertising content, there are other types of information, such as images and videos. Though we’ve controlled for the number of images in our research model, the design of the image and the video could still exert a significant effect on the ad responses from the consumers. Future research can apply more advanced image processing techniques to explore the impact of image design in in-feed native advertising (Rodgers, 2021). Second, given our computing capacity and the explanatory nature of this research, we’ve only tracked a relatively small amount of consumers from the server. Future research focusing on ad-response prediction can apply a larger dataset to fit a prediction model. The external validity of our research model still needs to be examined based on more behavioral data from the consumers. More importantly, this research only provides the general principles behind this dynamic targeting approach in the in-feed native advertising. Given the rich data of consumers’ online behaviors in those emerging news feed platforms, future research can explore other possibilities of “smart” content organizing and delivery. Such work will contribute to developing big data and AI (artificial intelligence) techniques (Rodgers, 2021) in the advertising industry.

## Data Availability Statement

The original contributions presented in the study are included in the article/supplementary material, further inquiries can be directed to the corresponding author/s.

## Author Contributions

BX wrote the first complete draft. HZ contributed additional writing and further analysis. Both authors conceptualized the manuscript, edited the manuscript, and approved the final version.

## Conflict of Interest

The authors declare that the research was conducted in the absence of any commercial or financial relationships that could be construed as a potential conflict of interest.

## Publisher’s Note

All claims expressed in this article are solely those of the authors and do not necessarily represent those of their affiliated organizations, or those of the publisher, the editors and the reviewers. Any product that may be evaluated in this article, or claim that may be made by its manufacturer, is not guaranteed or endorsed by the publisher.

## Footnotes

1
https://www.emarketer.com/content/us-native-advertising-2019
